# MRI techniques for immunotherapy monitoring

**DOI:** 10.1136/jitc-2022-004708

**Published:** 2022-09-19

**Authors:** Doreen Lau, Pippa G Corrie, Ferdia A Gallagher

**Affiliations:** 1 Centre for Immuno-Oncology, University of Oxford, Oxford, UK; 2 Department of Oncology, Addenbrooke’s Hospital, Cambridge, UK; 3 Department of Radiology, University of Cambridge, Cambridge, UK; 4 Cancer Research UK Cambridge Centre, University of Cambridge, Cambridge, UK

**Keywords:** Immunotherapy, Tumor Biomarkers, Immunologic Techniques

## Abstract

MRI is a widely available clinical tool for cancer diagnosis and treatment monitoring. MRI provides excellent soft tissue imaging, using a wide range of contrast mechanisms, and can non-invasively detect tissue metabolites. These approaches can be used to distinguish cancer from normal tissues, to stratify tumor aggressiveness, and to identify changes within both the tumor and its microenvironment in response to therapy. In this review, the role of MRI in immunotherapy monitoring will be discussed and how it could be utilized in the future to address some of the unique clinical questions that arise from immunotherapy. For example, MRI could play a role in identifying pseudoprogression, mixed response, T cell infiltration, cell tracking, and some of the characteristic immune-related adverse events associated with these agents. The factors to be considered when developing MRI imaging biomarkers for immunotherapy will be reviewed. Finally, the advantages and limitations of each approach will be discussed, as well as the challenges for future clinical translation into routine clinical care. Given the increasing use of immunotherapy in a wide range of cancers and the ability of MRI to detect the microstructural and functional changes associated with successful response to immunotherapy, the technique has great potential for more widespread and routine use in the future for these applications.

## Introduction

Harnessing the immune system to fight cancer using immunotherapy has recently emerged as a promising treatment for advanced cancers. Various interventions such as immune checkpoint inhibitors (ICI), anticancer vaccines, oncolytic viral therapy, and engineered chimeric antigen receptor (CAR) T cells have been developed to boost tumor immunity.^1^ While immunotherapy is increasingly being adopted into routine clinical practice, only a minority of treated patients currently benefit from these novel and costly agents. Therefore, there is an unmet need for effective clinical tools to predict outcomes early in the treatment pathway.

CT is the most widespread and routine imaging modality for response assessment following most cancer treatments including immunotherapy, particularly outside of the central nervous system. Biomarkers derived from whole blood sampling and invasive tumor biopsy are being explored but may be affected by sampling bias and may not reveal the full extent of the spatiotemporal changes that occur during treatment, or the natural history of the tumor.^2^ In contrast, imaging offers a non-invasive tool to assess the interpatient, intertumoral (intermetastatic), and intratumoral heterogeneity that is frequently seen with these agents in both space and time.^3^ A further level of heterogeneity is observed where the differential response of individual lesions may result in variable size changes in measurable metastases after immunotherapy.^4 5^ The identification of all these multilayered tumor heterogeneities is the key strength of imaging for immunotherapy monitoring.

Imaging may play a role in identifying true progression from pseudoprogression: the latter occurs in a small proportion of patients receiving ICIs, whereby the tumor initially enlarges before regressing in size. This was first described in patients with metastatic melanoma receiving ipilimumab^4^ and is the reason why the first time point for response assessment on ICIs is delayed until 12 weeks. Pseudoprogression may be the result of T cell infiltration and proliferation but is difficult to distinguish from true tumor progression using size measurements alone on conventional CT but has the potential to be identified by more specific functional and molecular imaging techniques.^4^ Related challenges for assessing response to ICIs include hyperprogressing lesions and dissociated or mixed response between lesions, which occurs in some patients on these agents.^6^ There is an unmet clinical need to develop tools for prediction of treatment response and early differentiation of treatment resistance from successful response.^7^

A longer-term key question is to more accurately characterize those patients who go on to develop durable response to therapy and long-term remission, as this has important implications for clinical management including balancing the need for treatment with toxicity risks, determining when therapy should be escalated, or if alternative treatment should be considered. Finally, determining the appropriate duration of long-term therapy and early identification of recurrent disease are other important considerations.

### Molecular imaging in cancer immunotherapy

Molecular imaging enables the visualization, characterization, and quantification of biological processes at the molecular and cellular levels in humans and other living systems.^8^ Imaging techniques such as MRI, single photon emission computed tomography (SPECT) and positron emission tomography (PET) are routine clinical tools for the non-invasive detection of tissue microstructure, function, and biochemical activities.^9 10^ Fluorine-18 fluorodeoxyglucose ([^18^F]FDG) PET is a widely used molecular imaging approach for cancer detection, staging, and monitoring of treatment response in patients, including immunotherapy.^11^ The rationale for using [^18^F]FDG as a glucose analog is based on the Warburg effect where cancer cells commonly exhibit high glucose uptake due to both increased glycolysis and ATP demand.^12^ Although [^18^F]FDG PET/CT is very sensitive for detecting distant metastases, the technique may demonstrate false-positive findings from physiological uptake as well as inflammation or infection and is therefore non-specific.^13 14^ [^18^F]FDG uptake in the tumor microenvironment often represents cancer cell uptake, but can also be due to infiltrating leukocytes which are also metabolically active, presenting a particular problem for the assessment of response to immunotherapy.^11^ For example, the infiltration of leukocytes into tumors such as metastatic melanoma following immunotherapy can produce a paradoxical increase in FDG uptake despite the successful response to therapy, which is known as the flare phenomenon. As a result, follow-up imaging must be conducted after this inflammatory response has resolved to confirm the true response of the tumor to treatment.^15^ [^18^F]FDG imaging in the prostate and brain are also limited due to high background physiological uptake by the surrounding tissues.^16 17^

Fluorine-18 fluorothymidine ([^18^F]FLT) is a radiolabeled analog of thymidine and marker of cell proliferation. It has been used for monitoring the proliferation of antigen-specific T cells in the lymph nodes of patients with melanoma following dendritic cell vaccination.^18^ Accumulation of [^18^F]FLT in tumors is related to thymidine kinase 1 enzymatic activity during the S-phase thymidine salvage and the de novo DNA synthesis pathways of the cell cycle.^19^ However, the measurement of cell proliferation in the setting of a malignant disease is not specific to activated T cells nor cancer cells. This was evident in a [^18^F]FLT PET study of patients with melanoma following 2 months of tremelimumab treatment (anti-cytotoxic T-lymphocyte antigen-4 (CTLA-4)) which showed no significant difference in [^18^F]FLT uptake within the tumors. An increase in [^18^F]FLT uptake was nevertheless detected in the spleens of 7 out of 10 patients, possibly due to treatment-induced T cell proliferation within the secondary lymphoid organs.^20^ Therefore, although they represent very sensitive approaches, immunotherapy monitoring using [^18^F]FDG and [^18^F]FLT PET has limitations including the difficulty in differentiating tumor progression from local inflammatory response based on single measurements of glucose metabolism or thymidine uptake.^20 21^

Newer radiotracers have been developed in recent years for immunotherapy monitoring in preclinical and clinical experimental medicine studies. Radiolabeled antibodies targeting CTLA-4, programmed cell death protein-1 (PD-1), programmed death ligand-1 (PD-L1), lymphocyte activation gene-3 (LAG3) and tumor necrosis factor receptor superfamily member 4 (TNFRSF4 or OX40) have showed potential in imaging drug biodistribution, and to some extent, the functional activity of leukocytes.^22–26^ Although zirconium-89 radiolabeled antibodies targeting CD8, PD-1 and PD-L1 have been developed for first-in-human trials,^27–30^ the optimal tracer dose for delivery into patients has yet to be established. The scan duration for many of these agents is long which renders translation to routine clinical application difficult but they have an important role in experimental medicine studies; in contrast, shorter-lived radioisotopes such as gallium-68 could potentially be used in the future for imaging antibody fragments or peptides relevant to immunotherapy targets.^31^ These novel radiolabeled approaches are still in early phase development and cannot be easily implemented into the clinic for routine diagnosis. In addition, the typical cost of a standard [^18^F]FDG PET/CT imaging study is greater than that for an MRI, therefore the cost-benefit of using these expensive imaging tests has to be carefully considered.^32^ PET is not suitable for frequent follow-up imaging and scanning of pediatric or pregnant patients due to the significant exposure to ionizing radiation. A typical PET scan could involve about three to five times as much radiation compared with the naturally occurring background radiation.^33^ Furthermore, PET does not provide any information on downstream metabolic products or compartmentalization of the signal within the tissue and has relatively low spatial resolution.^34^

MRI is a widely available clinical imaging tool with many strengths and potential to address some of the clinical challenges in assessing response to immunotherapy while overcoming many of the limitations of nuclear medicine imaging techniques as outlined above. MRI utilizes a range of sequences to probe tissue anatomy, microstructure, physiology, and function, generating excellent soft tissue contrast at high spatial resolution throughout the body. Various sequences can be used together in a multiparametric approach to produce a detailed imaging phenotype of the tumor microenvironment. Many of these sequences can be used to derive quantitative maps which either directly or indirectly probe fundamental biology and changes in the tumor microenvironment following treatment.

MRI is particularly suitable for functional and molecular imaging, as many of the measured parameters used to produce contrast in the images are dependent on the local chemical environment and therefore report on important biological properties of the tissues; examples of these include the spin–lattice relaxation (T_1_) and spin–spin relaxation (T_2_) times of the protons (^1^H) present within the water and fat in the tissue.^35^ MR spectroscopy (MRS) is based on the principle that nuclei within a molecule resonate in a magnetic field at a frequency which is dependent on the chemical structure of the molecule and can therefore be used to non-invasively identify and quantify individual tissue metabolites.^36^ A large number of MRI techniques have been developed over the years for probing the tumor immune microenvironment and monitoring treatment response to cancer immunotherapy and immune-related adverse events and are summarized in online supplemental table S1. These range from imaging microstructural and functional changes in the tumor microenvironment to molecular imaging approaches for detecting cellular and biochemical changes in tumors.

10.1136/jitc-2022-004708.supp2 Supplementary data

MRI is currently used in routine clinical practice to monitor the anatomical changes that occur following immunotherapy and is included in many of the Response Evaluation Criteria in Solid Tumors (RECIST) including the immune-related guidelines iRECIST,^37^ ^38^ and the Immunotherapy Response Assessment in Neuro-Oncology (iRANO) for monitoring response in tumors of the central nervous system, such as glioblastoma.^39–41^ This review will highlight key functional and molecular MRI approaches in addition to anatomical measures of tumor size for probing the tumor microenvironment and monitoring immunotherapy in patients, promising preclinical approaches, and the challenges in translating these methods into clinical practice. A list of abbreviations of the technical terms described in this review are found in online supplemental table S2.

10.1136/jitc-2022-004708.supp3 Supplementary data

### Imaging tumor cellularity

Diffusion-weighted imaging (DWI) is a non-invasive clinical tool commonly used for evaluating tumor cellularity and tissue microstructure. The technique is based on detecting the Brownian movement of water molecules in tissues. The method usually employs magnetic field gradients and a spin-echo MR sequence to probe these small molecular movements, which are approximately 20–30 µm in the typical 50–100 ms timescale of DWI.^42^ Tissue water molecules exhibit high diffusion in an unrestricted environment as is present in free fluid or necrotic tissues. In solid tumors, and in the presence of inflammation, increased cellular density reduces the extracellular space and increases the intracellular volume, resulting in restricted diffusion. The apparent diffusion coefficient (*ADC*) for water is used to quantify this diffusion and is calculated using different diffusion-weightings, or *b*-values, to derive metrics of diffusion behavior in tissues, generating quantitative maps which can be used as a surrogate biomarker for tumor cellularity.^36 43^

Low *ADC* measurements (equivalent to high signal on DWI) are often detected in cancerous tissues and have generally been attributed to increased cell density.^44^ Other factors such as the shape and size of intracellular spaces, tortuosity of the extracellular spaces, extracellular fibrosis and tumor necrosis may contribute to *ADC*.^36^ Importantly for measuring response to ICIs, cellular trafficking into the tumor microenvironment has an impact on diffusion as cancer cells are typically larger (10–20 µm in diameter) than naïve and activated T cells (5–10 µm) although often slightly smaller than macrophages (21 µm).^45 46^ Therefore, bulk cellular alterations during cancer immunotherapy may be detectable using DWI, whereby marked leukocyte infiltration may lead to reduced extracellular volume within tumors, restricted diffusion, and lower *ADC* during the initial phase of T cell migration, activation, and proliferation. Conversely, the latent effect of cytotoxic T cell killing of cancer may lead to an increase in the extracellular space within tumors, less restriction of water diffusion, and a higher *ADC*.^47^ There are a number of advanced DWI approaches which have been developed, including diffusion kurtosis imaging (DKI) for detecting tissue heterogeneity based on the non-Gaussian movement of water within the heterogeneous tumor microenvironment.^5 48^ In summary, DWI may be a very versatile, although non-specific tool, to evaluate overall cellular alterations within tumors during immunotherapy.

**Figure 1:**
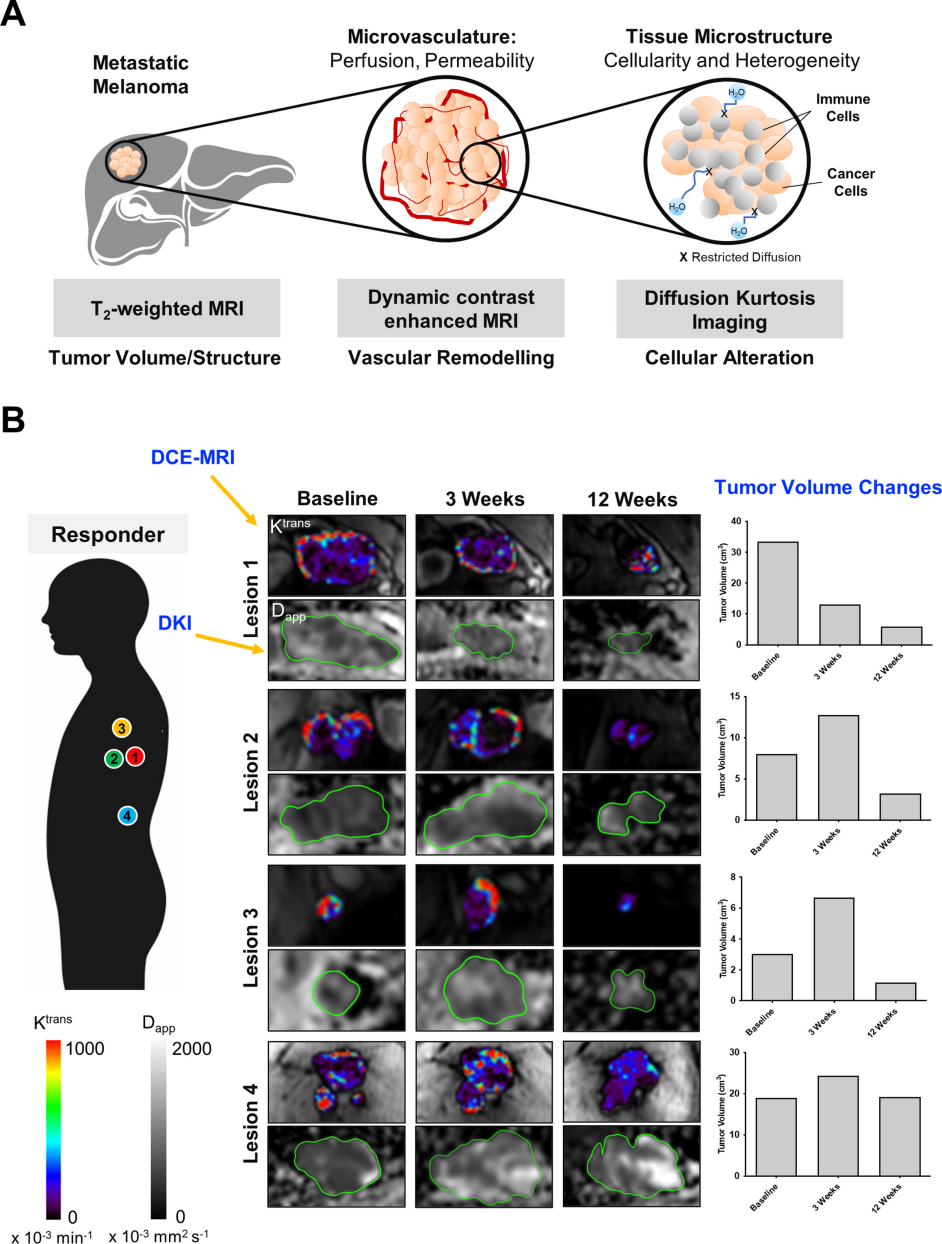
Multiparametric MRI for monitoring cancer immunotherapy. (A) Schematic diagram of the multiparametric MRI approach used for investigating changes in tumor volume (T_2_-weighted MRI), vascular permeability (DCE-MRI), tumor cellularity and heterogeneity (DKI) in patients with metastatic melanoma receiving immune checkpoint inhibitors. (B) Intertumoral differences in treatment response, vascular permeability, and cellularity were detected in a patient with metastatic melanoma during the first 12 weeks of treatment with nivolumab. Quantitative maps of tumor vascular permeability (*K^trans^*) and tumor cellularity (*D_app_*) were shown with corresponding changes in tumor volume measured on T_2_-weighted MRI. Images reproduced with permission from Lau *et al*.^5^ D_app_, apparent diffusivity; DCE, dynamic contrast-enhanced; DKI, diffusion kurtosis imaging; K^trans^, the volume transfer constant from the blood plasma to the extravascular tumor interstitial space.

Diffusion-weighted MRI techniques have been widely used to monitor response to cancer treatment in general, and more recently have been applied in immunotherapy trials.^5 49 50^ For example, a recent study of patients with glioblastoma treated with a dendritic cell vaccine and standard-of-care therapy demonstrated a higher pretreatment tumor *ADC* in the responders compared with the non-responders.^49^ There was a significant decrease in tumor *ADC* in the responders after 8 weeks of treatment which persisted during immunotherapy suggesting that leukocyte infiltration may have played a role, and this correlated significantly with longer progression-free survival. In contrast, several other researchers have reported an increase in tumor *ADC* following treatment with T cell-directed immunotherapy which may correspond to the later effects of cell death: a ≥1-fold increase in *ADC* at 5 weeks following immunostimulatory adenoviral CD40L gene therapy in previously treated ocular melanoma was shown to be a better predictor of objective survival than metabolic changes detected on [^18^F]FDG PET and tumor size changes detected on MRI.^50^ Similarly, in a recent multiparametric MRI study (figure 1) on treatment-naïve patients with metastatic melanoma, a significant increase in median apparent diffusivity (*D_app_*, equivalent to *ADC*) measured on DKI was detected in both responding and pseudoprogressive lesions as early as 3 weeks following immune checkpoint blockade.^5^ Treatment-induced changes in tumor cellularity detected on DKI was found to be independent of the tumor volume. Tumor heterogeneity (as measured by apparent kurtosis *K_app_* on DKI) was found to be consistently higher in the pseudoprogressive and true progressive lesions, compared with the responding lesions throughout the first 12 weeks of treatment possibly due to cellular alterations. These changes detected on DKI preceded tumor regression and vascularity changes detected on dynamic contrast-enhanced MRI.

However, despite the power of DWI as a non-invasive technique, the measurement of restricted diffusion and cellularity is not specific to cancer or leukocytes. Although low ADC (or D_app_) values have generally been attributed to increased tumor cell density, other factors also contribute to the low ADC measurements as outlined above.^36 44^ A significant complication when using DWI for assessment of successful response to immunotherapy is the increased cellularity (and hence restricted diffusion) that may arise from leukocyte infiltration into tumors, which may be hard to distinguish from true tumor cellularity. Therefore, DWI forms one of several MRI approaches to disentangle the complex cellular biological processes that develop during treatment and may offer additional cellular information when used in combination with other techniques in a multiparametric or hybrid imaging approach for evaluating immunotherapy response.

### Imaging tumor vascular permeability and inflammation

The tumor vasculature plays diverse roles in regulating tumor growth, metastasis, and leukocyte trafficking in and out of the tumor microenvironment. T cell migration and infiltration into tumors requires transport via the hematological and lymphatic routes, chemokine signaling, and the expression of cell adhesion molecules on endothelial cells and activated leukocytes, for example, selectins, intercellular adhesion molecule 1 (ICAM-1) and vascular cell adhesion molecule 1 (VCAM-1).^51^ Combined treatment using anti-VEGFR2 and anti-PD-L1 has been shown to induce T cell activation and infiltration in responding tumors by promoting blood vessel normalization in preclinical models of breast and pancreatic cancer.^52^ Hence, imaging tumor vascularity and inflammation may provide a surrogate biomarker of tumor vasculature response to immunotherapy.

Malignant tumors typically undergo angiogenesis and exhibit immature, tortuous, and leaky blood vessels that are permeable to intravascular contrast agents, unlike normal blood vessels. Gadolinium chelates are used to provide vascular contrast enhancement on T_1_-weighted MRI sequences and are used as part of routine MRI to provide qualitative or semi-quantitative measures of vascularity. Dynamic contrast-enhanced MRI (DCE-MRI) provides more quantitative measurements of vascular permeability and is often used in the context of experimental medicine studies for evaluating treatment response to angiogenesis inhibitors.^53 54^ The technique is performed by acquiring a series of T_1_-weighted images following the bolus injection of an intravascular contrast agent and using pharmacokinetic modeling to derive quantitative measurements of tissue perfusion and vascular permeability such as the volume transfer constant from the blood plasma to the extravascular tumor interstitial space (*K^trans^*), fractional volume of the interstitial space (*v_e_*), and fractional volume of the blood plasma (*v_p_*). A recent clinical study in patients with previously irradiated melanoma brain metastases showed lower *K^trans^* and *v_p_* in the pseudoprogressive lesions following 9 weeks of ipilimumab (anti-CTLA-4).^55^ In a separate study on treatment-naïve patients, a gradual decrease in *K^trans^*, *v_e_*, and *v_p_* was also detected in the responding and pseudoprogressive melanoma metastases over 12 weeks of pembrolizumab (anti-PD-1) monotherapy or combined ipilimumab (anti-CTLA-4) and nivolumab (anti-PD-1) therapy.^5^ Nevertheless, a significant decrease in tumor vascular permeability was only detectable following a reduction in tumor volume and tumor cell loss, showing that vascular normalization occurs relatively late in response to immunotherapy and after cell death has begun. This contrasts with antiangiogenic therapy in human melanoma where the treatment effects are targeted directly towards the vascular network and are generally non-cytotoxic.^56^ These studies provide evidence that microstructural and functional measurements of the tumor vasculature using DCE-MRI may have utility in detecting successful treatment response to ICIs, but only at the later time points following immune cytotoxic killing, tumor regression, vascular normalization and shutdown, and are best visualized in a multiparametric approach.

Preclinically, *in vivo* expression of biomarkers related to vascular inflammation, for example, VCAM-1, ICAM-1 and selectins may be detectable on MRI using antibodies conjugated to iron oxide nanoparticles or bifunctional chelates of paramagnetic or superparamagnetic metal cations.^57–59^ For instance, antibodies targeting VCAM-1, a cell surface glycoprotein essential for leukocyte adhesion to the endothelium through α4β1 integrin binding have been conjugated to microparticles of iron oxide (MPIO) for non-invasive imaging and quantification of VCAM-1 density in atherosclerosis, brain micrometastases, as well as models of cancer immunotherapy.^57 59 60^ Binding of VCAM-1 MPIOs to their target create local magnetic field inhomogeneities detected as hypointense signals on T_2_^*^-weighted MRI. A recent study demonstrated the presence of more hypointense signals (reflecting higher VCAM-1 expression) and greater T cell density on histology in pretreated EL4 mouse lymphoma and CT26 mouse colorectal carcinoma tumors, compared with the more immunologically cold B16 mouse melanoma.^57^ Imaging VCAM-1 density before treatment initiation in an immunologically cold MC38 mouse colorectal carcinoma model showed that the MRI measurement was predictive of response to PD-L1 blockade as more hypointense signal was associated with tumor growth inhibition following 2 weeks of treatment.^57^ Dual-targeted MPIOs (for both VCAM-1 and P-selectin) have been developed for visualizing endothelial activation in atherosclerosis and to mimic the *in vivo* dynamics of initial leukocyte rolling which is selectin-mediated and subsequent leukocyte adhesion through integrin binding.^61^

Antibody-conjugated MPIOs offer promise for intravascular imaging as these large particles (>1 µm in diameter) are less susceptible to extravasation or non-specific uptake by endothelial cells compared with ultrasmall superparamagnetic iron oxide (USPIO) nanoparticles (<0.05 µm). MPIOs have a short circulatory half-life (<5 min), a high iron payload that is in orders of magnitude greater than USPIO, induce strong contrast changes on T_2_^*^-weighted MRI and field inhomogeneities extending ~50-times the diameter of the particle. The larger surface area of MPIO (2–12 µm^2^) compared with USPIO (0.005–0.03 µm^2^) enables greater ligand valency when modifying MPIO for molecular targeting, substantially improving the binding affinities through multivalent effects.^62^ However, clinical translation of MPIOs for imaging vascular inflammation generally has been challenging because the current MPIOs used in research contain a non-biodegradable polyurethane coating and tend to accumulate and persist in the liver and spleen due to the reticuloendothelial route of clearance. In addition, full-length antibodies with intact F_c_ region or from a murine origin may be immunogenic.^63^ Tumors tend to have irregular blood vessels and inefficient lymphatic drainage; hence relatively large imaging agents tend to accumulate non-specifically in tumors due to enhanced permeability and retention effects.^64^ Therefore, efforts have been made in recent years to develop peptidase-degradable or biocompatible polymer encapsulated MPIOs for human use.^62 65^ The adoption of humanized antibodies, F_c_-silent antibody fragments or peptides for developing molecular MRI contrast agents is paramount for clinical translation.^66^

### Imaging tumor metabolism

Metabolic reprogramming is one of the hallmarks of cancer.^67^ Tumor metabolism plays an important role in governing the function and phenotypic plasticity of leukocytes.^68^ Malignant tumors typically exhibit a high energy demand and increased expression of glucose transporters such as GLUT1, enabling increased glucose uptake, which helps to fuel rapid cellular proliferation. This results in increased production of metabolic by-products such as lactic acid, which together with hypoxia and the high interstitial fluid pressure found within tumors, contributes to an immunosuppressive tumor microenvironment.^69^ Furthermore, increased tumor glycolysis restricts extracellular glucose availability to T cells.^69^ Lactic acid is also produced by proliferating T cells and in turn may inhibit human CD8^+^ T cell activation, proliferation, and effector functions,^70^ while neutralization of tumor acidosis using bicarbonate treatment improves response to ICIs and adoptive T cell therapy in preclinical models.^71^ Imaging metabolism may therefore be important for probing the immune compartment within tumors and treatment-induced changes. The most established technique is [^18^F]FDG PET: a complete metabolic response (CMR) with [^18^F]FDG uptake 1 year after commencing anti-PD-1 therapy is associated with excellent progression-free survival compared with those patients who do not show CMR.^72^ Although [^18^F]FDG has been a very successful approach for reporting local glucose uptake in tumors, it provides no direct information about glycolytic flux or oxidative phosphorylation. Moreover, it is not known whether [^18^F]FDG can detect early response to treatment as it can be particularly difficult to distinguish tumor metabolism from glucose uptake associated with leukocyte infiltration after the initial introduction of immunotherapy.^73^

Several MRI methods have been employed to date for probing tumor metabolism such as ^1^H-MRS described above, both to measure steady-state levels of tissue metabolites and how these change with treatment.^74^ Other methods include GlucoCEST which has been translated into human imaging and uses chemical exchange saturation transfer (CEST) to selectively detect glucose concentration within tissue after oral or intravenous administration of unlabeled glucose, although it does not distinguish this from downstream metabolites.^75^ More recently, deuterium (^2^H) metabolic imaging has emerged as a potentially important technique to evaluate the metabolism of [6,6’-^2^H_2_]glucose administered intravenously in animals or orally in humans and the subsequent production of both ^2^H-lactate or ^2^H-glutamine+glutamate (^2^H-Glx).^76^ This has been applied to brain tumors to date^76^ and has recently been translated to clinical field strengths showing its potential to evaluate metabolism in a wide range of cancers in the clinic.^77^

**Figure 2:**
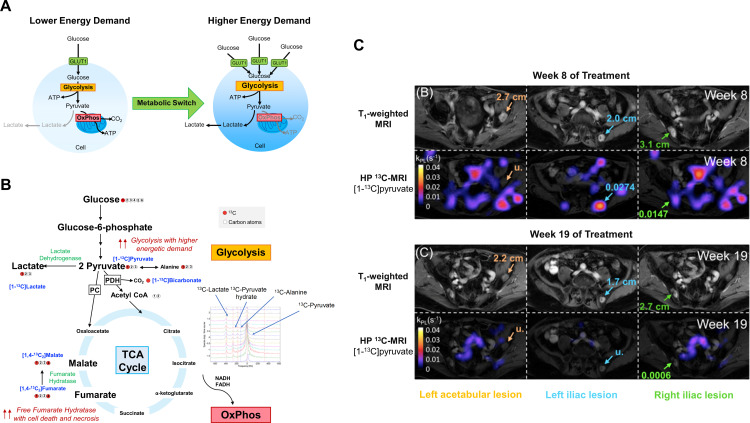
Imaging glucose metabolism with HP ^13^C-MRI. (A) An increased expression of glucose transporters, for example, GLUT1 and elevated glycolysis is seen in cells with higher energy demand, for example, cancer cells and activated T cells. (B) Several ^13^C-labeled imaging probes have been developed for evaluating different downstream processes of the glucose metabolism pathway. These include [1-^13^C]pyruvate for imaging the kinetics of pyruvate-to-lactate conversion,^78^ ^13^C-labeled bicarbonate (H^13^CO_3_^−^) for detecting tumor pH *in vivo*,^79^ and increased production of [1,4-^13^C_2_]malate from the administered [1,4-^13^C_2_]fumarate as a surrogate biomarker of cell necrosis or tumor cell death in response to treatment.^80^ (C) HP ^13^C-MRI of a patient with prostate cancer showed a marked decrease in [1-^13^C]pyruvate to [1-^13^C]lactate conversion, with a corresponding reduction in tumor size, in the three bone metastases (left acetabular, left and right iliac lesions) between week 8 and 19 following pembrolizumab treatment. Images adapted and reproduced with permission from de Kouchkovsky *et al*.^87^ (A) and (B) are graphics created by the author (DL). ATP, adenosine triphosphate; NADH, reduced nicotinamide adenine dinucleotide; FADH, reduced flavin adenine dinucleotide; TCA, tricarboxylic acid; PC, pyruvate carboxylase ; PDH, pyruvate dehydrogenase.

Pyruvate is a breakdown product of glucose sitting at an important metabolic junction between intramitochondrial oxidative metabolism and cytosolic reductive metabolism, which offers an alternative approach to imaging glucose metabolism. The conversion of pyruvate to lactate in tissues, under the action of lactate dehydrogenase, can be imaged using the emerging clinical tool of hyperpolarized carbon-13 MRI (HP ^13^C-MRI).^34^ By applying the technique of dynamic nuclear polarization to ^13^C-labeled endogenous metabolites, the signal-to-noise ratio can be increased by 10,000–100,000 fold, sufficient to allow the spatial distribution of the hyperpolarized ^13^C-labeled imaging probe to be detected after intravenous injection, and the detection of the immediate metabolites formed from it dynamically over time.^78^ Several ^13^C-labeled imaging probes have been developed over the years for interrogating different aspects of the glucose metabolism pathway including [1-^13^C]pyruvate for imaging the kinetics of pyruvate-to-lactate conversion,^78^ The ^13^C-labeled bicarbonate (H^13^CO_3_^−^) for detecting tumor pH *in vivo*,^79^ and increased production of [1,4-^13^C_2_]malate from the administered [1,4-^13^C_2_]fumarate as a surrogate biomarker of cell necrosis or tumor cell death in response to treatment^80^ (figure 2). Many of these could have potential for monitoring leukocyte infiltration and metabolism, with [1-^13^C]pyruvate the main molecule used clinically.

[1-^13^C]Pyruvate has been evaluated in preclinical models and patients including glioblastoma, breast, kidney and prostate cancer.^81–84^ In preclinical studies, ICI-resistant 3I-F4 melanoma tumors derived from B16 melanoma exhibited a hypermetabolic state whereby both glycolysis and oxidative phosphorylation were upregulated.^85^ The treatment-resistant 3I-F4 tumors were found to have a significantly higher rate of pyruvate-to-lactate conversion *in vivo* on hyperpolarized [1-^13^C]pyruvate MRI compared with the more treatment-sensitive B16 melanoma. These treatment-resistant tumors were able to thrive under hypoxic conditions and this metabolic adaptation created a hostile tumor microenvironment which inhibited effector T cell function and proliferation. In a separate study, a multiparametric imaging approach using DCE-MRI, hyperpolarized [1-^13^C]pyruvate and [1,4-^13^C_2_]fumarate MRI was used to simultaneously detect early changes in tumor vascular permeability and perfusion, glycolysis, and necrotic cell death in response to combined anti-CTLA-4 and anti-PD-L1 therapy.^86^ Following successful response to treatment, a significant increase in [1,4-^13^C_2_]fumarate to [1,4-^13^C_2_]malate conversion indicating tumor cell death, and tumor vascular permeability and perfusion (as measured by *K^trans^*) was detected in MC38 colorectal tumors. These changes were not significant in the less treatment-sensitive B16-F10 melanoma. However, a significant decrease in pyruvate-to-lactate conversion was detected in B16-F10 tumors following treatment, but not in the more treatment-sensitive MC38 colorectal tumors. This suggested that B16-F10 is more dependent on glycolysis for energy production than MC38.

The metabolic response to immune checkpoint blockade using HP ^13^C-MRI was recently reported in a case example of a patient with prostate cancer treated with pembrolizumab. A decrease in pyruvate-to-lactate conversion, with corresponding reduction in tumor volume, was detectable between weeks 8 and 19 of treatment.^87^ HP ^13^C-MRI has also showed potential in detecting early metabolic response to neoadjuvant chemotherapy in breast cancer where a very early increase in lactate production was associated with complete pathological response at the later surgery. It is possible that this early increase in metabolism can be partly explained by leukocyte infiltration in the responding tumors although confirmation with pathology would be useful in future studies.^88^ [1-^13^C]Pyruvate has also been used to distinguish metabolism within the epithelial compartment from stromal metabolism in human prostate cancer^84^ and identifying this metabolic compartmentalization lends support to its potential role in the future for distinguishing immunometabolism within the tumor microenvironment. However, HP ^13^C-MRI is an emerging technique requiring dedicated hardware, imaging must be undertaken very rapidly after injection due to the rapid signal decay, and the method is not yet a routine clinical tool. Furthermore, in general, metabolic imaging approaches acquire images at relatively low spatial resolution compared with conventional anatomical MRI, which limits the size of the lesions that can be successfully monitored for treatment effects.^89^

In summary, MRI provides a wide range of methods to metabolically phenotype tumors. Despite the relative lack of sensitivity of MRI compared with radionuclide imaging, its major strength is the ability to identify metabolites, distinguish separate metabolic pathways and tracking dynamic changes in metabolism.

### Tracking labeled leukocytes

Cellular immunotherapy and other immune-directed treatments are increasingly used for modulating specific leukocyte subpopulations to enhance tumor immunity. Therapeutic cells such as CAR T cells and antigen-primed dendritic cells (DC) have been developed for targeting tumor-associated antigens.^90^ Immunomodulatory drugs in the form of monoclonal/bispecific antibodies and small molecules have been used for redirecting or altering the phenotypes and functions of immunosuppressive cell types such as myeloid derived suppressor cells and regulatory T cells.^91 92^ Treatment efficacy often depends on the efficient targeting and delivery of these specific leukocytes to the tumor microenvironment. Non-invasive methods to track the *in vivo* fate and biodistribution of leukocytes, and changes in their localization, density and persistence in tumors, secondary lymphoid organs and the whole body on a system level will be useful for optimizing the precise timing, dosing, and delivery of treatment.^93^

**Figure 3:**
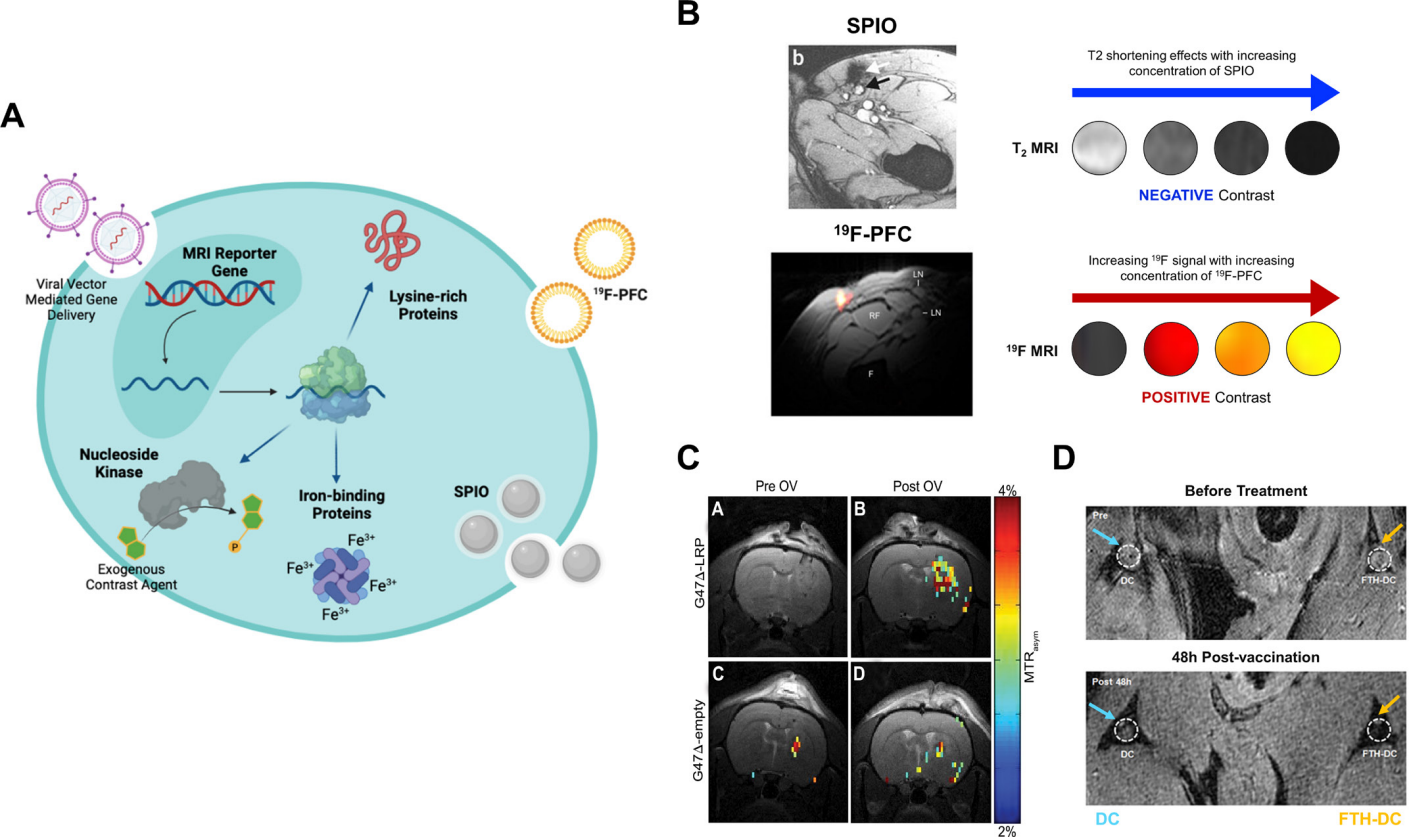
MRI tracking of leukocytes and viral vector-mediated gene therapy. (A) Graphical overview of direct and indirect cell labeling approaches with superparamagnetic iron oxide (SPIO) nanoparticles, ^19^F-perfluorocarbons (PFC), and examples of MRI reporter genes. (B) A comparison between the negative contrast (decreased signal) obtained with SPIO versus the positive contrast (increased signal) obtained with ^19^F-PFC labeling of human dendritic cells. (C) Chemical exchange saturation transfer MRI of lysine-rich protein (LRP) concentration in rat glioma tumors before and at 8 hours following G47Δ oncolytic viral (OV) therapy showed higher signal on the magnetization transfer ratio asymmetry (MTR_asym_) maps of tumors injected with G47Δ-LRP but not the empty vector. (D) T_2_-weighted MRI showed signal loss or decreased T2 signal (orange arrow) at the popliteal lymph node near the footpad of a mouse at 48 hours following injection with dendritic cells expressing ferritin (FTH-DC). (A) is a graphic created by author (DL) using Biorender (publication licence AU24ELW1E5). Images in (B), (C) and (D) are reproduced with permission from Ahrens *et al*, Farrar *et al*, de Vries *et al* 110 123 170 and Kim *et al*.^124^

MRI tracking of leukocytes has been performed using direct or indirect labeling of cells with superparamagnetic iron oxide (SPIO) nanoparticles and Fluorine-19 (^19^F) perfluorocarbons, or genetic modification of cells with MRI reporter genes for longitudinal imaging (figure 3). SPIOs are iron oxide cores that can induce a local magnetic susceptibility effect which dephase the protons, reduces the signal intensity and creates negative contrast on T_2_-weighted and T_2_^*^-weighted images. Indirect cell labeling can be undertaken by administering the SPIOs intravenously which can be internalized by phagocytes (eg, macrophages and DCs) via phagocytic uptake or pinocytosis *in vivo*.^94 95^ SPIOs are the most widely used agents for MRI cell labeling due to their relative higher detection sensitivity on clinical ^1^H-MRI compared with ^19^F-MRI with 10^5^ DCs required in human lymph nodes for ^1^H detection compared with 10^6^–10^7^ with ^19^F-labeling.^94^ Ferumoxytol (Feraheme) is an ultrasmall SPIO (17–30 nm) and an iron supplement approved by the Food and Drug Administration for the treatment of anemia in chronic kidney disease and has been used for imaging inflammatory atherosclerotic plaques.^96^ This agent has been repurposed for the non-invasive detection of tumor-associated macrophages (TAMs) in both patients and preclinical models.^97–100^ A decrease in ferumoxytol signal has been detected in MMTV-PyMT breast tumors depleted of macrophages following treatment with macrophage colony-stimulating factor 1 monoclonal antibodies.^99^ In contrast, blockade of the integrin-associated protein CD47 on cancer cells and its interactions with the inhibitory receptor signal-regulatory protein alpha (SIRPα) on macrophages using anti-CD47 can reactivate TAMs to phagocytose cancer cells. An increase in ferumoxytol enhancement following treatment was detected in preclinical osteosarcoma models: this corresponded to a significantly higher density of F4/80^+^ macrophages and phagocytosis activity within tumors.^100^

Although ferumoxytol-enhanced MRI has demonstrated feasibility in detecting TAMs and is clinically available, measurements of T_2_^*^ signal can be compromised by the presence of endogenous iron, hemorrhage, deoxyhemoglobin, and inflammation within tumors. Hence, a significant difference in the precontrast and postcontrast images will be required to detect any changes in TAMs.^101^ The method is qualitative or semi-quantitative, as factors such as the non-linear relationship between the contrast concentration and T_2_^*^ relaxation time can affect measurements of SPIO distributions. Susceptibility artifacts that may arise from air-tissue interfaces, such as in the lung or brain sinuses, can result in an inaccurate estimation of T_2_^*^ relaxation, especially at very low concentrations of SPIO-labeled cells.^102^

^19^F-MRI may provide a more quantitative approach for tracking leukocytes.^103^ Although ^19^F has a slightly lower sensitivity compared with ^1^H (83% of ^1^H), the signal obtained from ^19^F-based imaging agents is highly specific due to the lack of endogenous or background ^19^F in biological tissues. This facilitates the detection of ^19^F-labeled cells as positive contrast on ^19^F-MRI, rather than negative T2 contrast changes on ^1^H-MRI with SPIOs.^104 105^ The ^19^F-labeled perfluorocarbons (PFC) have been used in patients and preclinical models for tracking leukocyte fate and biodistribution. PFCs are biochemically inert and lipophilic agents that can efficiently enter cells via passive uptake.^103^ They can be prepared into lipid nanoemulsions or be modified with synthetic polymers, for example, poly(lactide-co-glycolide) to improve their biocompatibility as imaging agents.^106^ If the leukocytes are directly labeled *ex vivo* before adoptive transfer, the number of cells that have migrated to tumors or other tissues of interest can be estimated from the ^19^F images by quantifying the ^19^F signal in the region of interest and reference tubes containing a known amount of ^19^F, and using ^19^F nuclear magnetic resonance (NMR) to determine the number of ^19^F spins per cell prior to injection.^107^

^19^F-PFC nanoemulsions have been used in several studies for imaging the dynamics of human leukocyte migration and tumor infiltration, for example, CAR T cells, natural killer (NK) cells, DCs and peripheral blood mononuclear cells.^108–110^ One notable example is the tracking of ^19^F-labeled human CAR T cells in severe combined immunodeficient mice subcutaneously implanted with EGFRvIII expressing human glioblastoma.^108^ Cell viability and proliferation status were retained in T cells labeled overnight with 10 mg/mL of a clinical-grade PFC (CS-1000, Celsense). Tissue biodistribution studies conducted at 2, 7, and 14 days post-treatment using ^19^F-NMR showed approximately twice as many CAR T cell homing to the tumors and spleens on Day 2. The apparent number of labeled CAR T cells detected in the tumors remained relatively stable on Day 7, which corresponded to a significantly slower tumor growth in CAR T cell treated mice compared with mice injected with naïve T cells. The ^19^F signal further reduced on Day 14, likely due to cell proliferation and label dilution or cell death and PFC clearance.

However, clinical translation of ^19^F-MRI has been limited by the inherent low sensitivity of the technique. This was seen in a study on patients with colorectal cancer using autologous DCs labeled with ^19^F-PFC to evaluate cellular migration to the draining lymph nodes for antigen presentation to T cells.^110^ An initial strong ^19^F signal was detected at the injection site on 3T MRI after intradermal administration of 10^7^ labeled DCs. However, the results obtained 24 hours later were modest; no signal was detected in the draining lymph nodes within the same field of view although a 50% loss in signal was detected at the injection site. This may be due to PFC clearance at the injection site following cell death, loss of signal due to cell division, inherent low sensitivity of ^19^F-MRI at clinical field strength MRI systems (1.5–3T) for detecting the migration of a small number of DCs, or a combination of all these factors.^103^ Future improvements to improve the detection sensitivity of ^19^F-MRI include employing advanced MRI hardware, improved imaging sequences and post-processing (eg, compressed-sensing, ultrashort echo-times), or imaging at higher magnetic field strength, for example, 7T in the research setting.^111–113^ The clinical application of ^19^F-MRI for immunotherapy monitoring requires specialized hardware and software approaches for image acquisition,^114^ and it is not yet a routine imaging tool. Furthermore, the engineered T cells commonly used in immunotherapy are weakly phagocytic and have a small cytoplasmic volume which poses a challenge for current intracellular labeling approaches in MRI such as ^19^F-PFC nanoemulsions, when compared with labeling phagocytes such as neutrophils; however, there have been recent advances in ^19^F probes such as the use of cell-penetrating peptides which show promise in labeling CAR T cells with enhanced ^19^F-MRI detection.^115^ The choice of cell types and imaging time points are crucial for direct labeling and should be carefully optimized for the specific application, as cell proliferation may lead to signal dilution over longer time periods. For example, neutrophils are terminally differentiated cells, hence the MRI signal from the labeled cells may not decrease as much due to cell proliferation unlike activated T cells.^116^ Alternatively, MRI reporter genes may be used for long-term tracking of leukocytes that undergo cell proliferation and differentiation.

### Reporter gene imaging

Reporter gene imaging is an indirect method for cellular detection based on imaging transgene expression. The approach involves the incorporation of reporter genes which express proteins (eg, membrane transporters, cell surface and intracellular receptors, or enzymes) that can be detected using specific probes or are based on the tissue contrast they generate.^117^ Reporter genes could serve as molecular beacons to signal the presence of therapeutic cells or incorporation of viral transgenes. They are only expressed by viable cells, and if stably incorporated into the genome could enable long-term follow-up on imaging.^118 119^

Several reporter genes have been employed for tracking leukocytes and assessing the efficacy of viral vector-mediated gene delivery. Most studies have involved the use of genetically encoded fluorescent proteins such as the green fluorescent protein (GFP) for tracking cellular kinetics and cell–cell interactions *in situ* using intravital microscopy, or on a whole-body level using bioluminescence imaging and radionuclide imaging.^120 121^ A wide range of MRI reporters have been developed for stem cell tracking and evaluating viral vector-mediated gene delivery in neurological, cardiac, and orthopedic applications,^122^ with potential for development in immuno-oncology. To date, only a few examples of MRI reporter gene imaging have been undertaken in preclinical cancer immunotherapy studies^123–125^ (figure 3).

Cationic polymers are biodegradable, artificial polypeptides that have been used for non-viral gene delivery in human cells, including T cells.^126^ They contain a number of rapidly exchanging amide protons which can produce an endogenous contrast on CEST MRI, potentially at micromolar concentrations, and eliminates the need for contrast administration to detect the reporter gene.^127 128^ One example is the lysine-rich protein (LRP) which comprises 200 lysine residues and has been used for CEST MRI-based reporter gene imaging of cancer cells, adenoviral gene transfer in heart failure, and oncolytic viral therapy.^129–131^ The LRP reporter gene has been engineered into G47Δ, a herpes simplex-derived oncolytic virus (HSV) currently being evaluated in clinical trials. Incorporation of the LRP reporter gene did not interfere with HSV replication in cancer cells and expression of LRP. MR imaging of rat glioma tumors at 9.4T showed a significant increase in CEST contrast enhancement in tumors infected with LRP-expressing HSV, compared with tumors infected with the control empty virus, and demonstrated tumor heterogeneity in viral spread.^123^ However, the applicability of this method for tracking cell and reporter gene imaging more generally in the clinic remains to be elucidated.

Ferritin has been utilized as an MRI gene reporter for tracking neural stem cells, detecting drug-inducible gene expression in cancer, and monitoring viral vector-mediated gene delivery.^132^ It is a metalloprotein ubiquitously expressed in most cells required for the uptake, storage, and controlled release of iron in living tissues.^133^ Cells overexpressing the heavy chain of ferritin (FTH) can intracellularly load and accumulate iron oxide cores, which are paramagnetic and can act as an endogenous contrast to affect the MR relaxation rates of water or other molecules, generating areas of hypointensity on T_2_-weighted and T_2_^*^-weighted images, analogous to those seen with SPIO labeling of cells. Mouse DCs have been transduced with the FTH and GFP reporter genes under a *myc* promoter to study their migration into lymph nodes.^124^ The transduced DCs exhibited similar *in vitro* proliferation, migratory capabilities, and expression of co-stimulatory molecules (CD40, CD80 and CD86) as seen in naïve DCs, but with increased iron storage capacity. T_2_^*^-weighted images obtained at 9.4T showed hypointensities within the popliteal lymph nodes of mice at 48 hours following injection of 10^7^ transduced DCs in the footpad. Histology showed the presence of GFP^+^ transduced DCs below the lymph node capsules and T cell zone, and the expression of CD25 (a marker for mature DCs involved in T cell binding and activation). The normal biodistribution of the transduced DCs were detected in the spleen, pancreatic, and mesenteric lymph nodes. These demonstrated feasibility in using ferritin for longitudinal tracking of DC-based vaccination and antigen presentation. However, ferritin is expressed endogenously in living tissues and is often elevated in the serum of patients with cancer.^134^ Marked differences in the pretreatment and post-treatment images of the lymph nodes following DC vaccination will be needed to detect any difference. Furthermore, tissue damage, oxidative stress, bleeding, and inflammation can elevate ferritin levels and contribute to the background signal on T_2_^*^-weighted images.^135^ Thus, the specificity and sensitivity of ferritin as an MRI reporter gene for longitudinal tracking of cellular immunotherapy requires further validation.

A recent study explored the use of a nucleoside kinase reporter gene with a companion imaging agent for tracking DCs in mice using CEST MRI.^125^ The nucleoside kinase *Drosophila melanogaster 2’-deoxynucleoside* (Dm-dNK) can phosphorylate and intracellularly trap all native deoxynucleosides and a wide range of synthetic nucleosides including the fluorescent nucleoside analog, *pyrrolo-2’-deoxycytidine* (pyrrolo-dC), which can be detected on CEST MRI and flow cytometry. Imaging studies conducted at 48 hours following DC vaccination at the footpad in mice showed significantly higher CEST contrast enhancement in the popliteal lymph nodes that received Dm-dNK transduced DCs compared with mice that received naïve DCs. Flow cytometric analysis showed that on average <10^4^ Dm-dNK transduced DCs accumulated pyrrolo-dC in each lymph node. These findings demonstrated sensitivity of the method in detecting DCs migration into draining lymph nodes at a preclinical level. However, an ideal reporter system needs to be biocompatible or endogenous for ease of translation into clinics. As Dm-dNK is a foreign protein from the fruit fly, further work is required to evaluate its immunogenicity and whether the expression of the foreign gene will affect survival of the transduced cells.

Non-immunogenic proteins such as the human organic anion transporting polypeptide (OATP) could be explored as MRI reporters for immuno-oncology studies.^136^ Different isoforms of OATP are endogenously expressed in the hepatobiliary system and are involved in the cellular uptake of small anions, including bilirubin in the liver and some nutrients from the small intestine. Expression of OATP in the transduced cells can be detected by an MRI contrast agent commonly used for liver imaging such as gadolinium ethoxybenzyl diethylenetriamine pentaacetic acid (Primovist) which can produce increased T_1_-weighted signal on MRI.^136 137^ Furthermore, OATP can be detected using radionuclide and fluorescent imaging agents.^136 137^ This combines the merits of hybrid imaging modalities, that is, pairing the greater sensitivity and molecular specificity of radionuclide imaging with the excellent soft tissue contrast and anatomical details of MRI for tracking leukocytes and viral vector-mediated gene delivery in non-hepatobiliary regions of the body.^138^

### Imaging immune-related adverse events

Systemic immune checkpoint blockade using ICIs have been approved for routine use to treat a growing list of cancers in early and advanced stages of the disease. ICIs may generate immune-related adverse events (irAEs), some of which can be both life-threatening and life-changing. These irAEs often result from systemic T cell reactivation in healthy tissues and can occur in any organ system. Severe or life-threatening irAEs (Grade ≥3) have been reported in 20–30% of patients receiving ipilimumab, and 10–15% of patients on anti-PD-1 treatment, with the highest incidence rate of 55% reported in patients undergoing combined anti-CTLA-4 and anti-PD-1 therapy.^139 140^ The more commonly affected organ systems are the skin, gastrointestinal tract, liver, and endocrine system. Emergency hospitalizations are needed for patients experiencing severe colitis, pneumonitis, rarer neurological events such as meningitis and Guillain-Barré syndrome, while treatment-related deaths have resulted mainly from myocarditis and colitis. Chronic debilitating toxicities can be problematic affecting joints, muscles, and vision, while there is increasing concern regarding the potential for long-term damage to patients receiving high-dose steroids and other immunosuppressive agents to manage these irAEs. The increasing use of ICIs has unveiled an unmet need for non-invasive imaging tools to diagnose and monitor irAEs rapidly and specifically.

**Figure 4:**
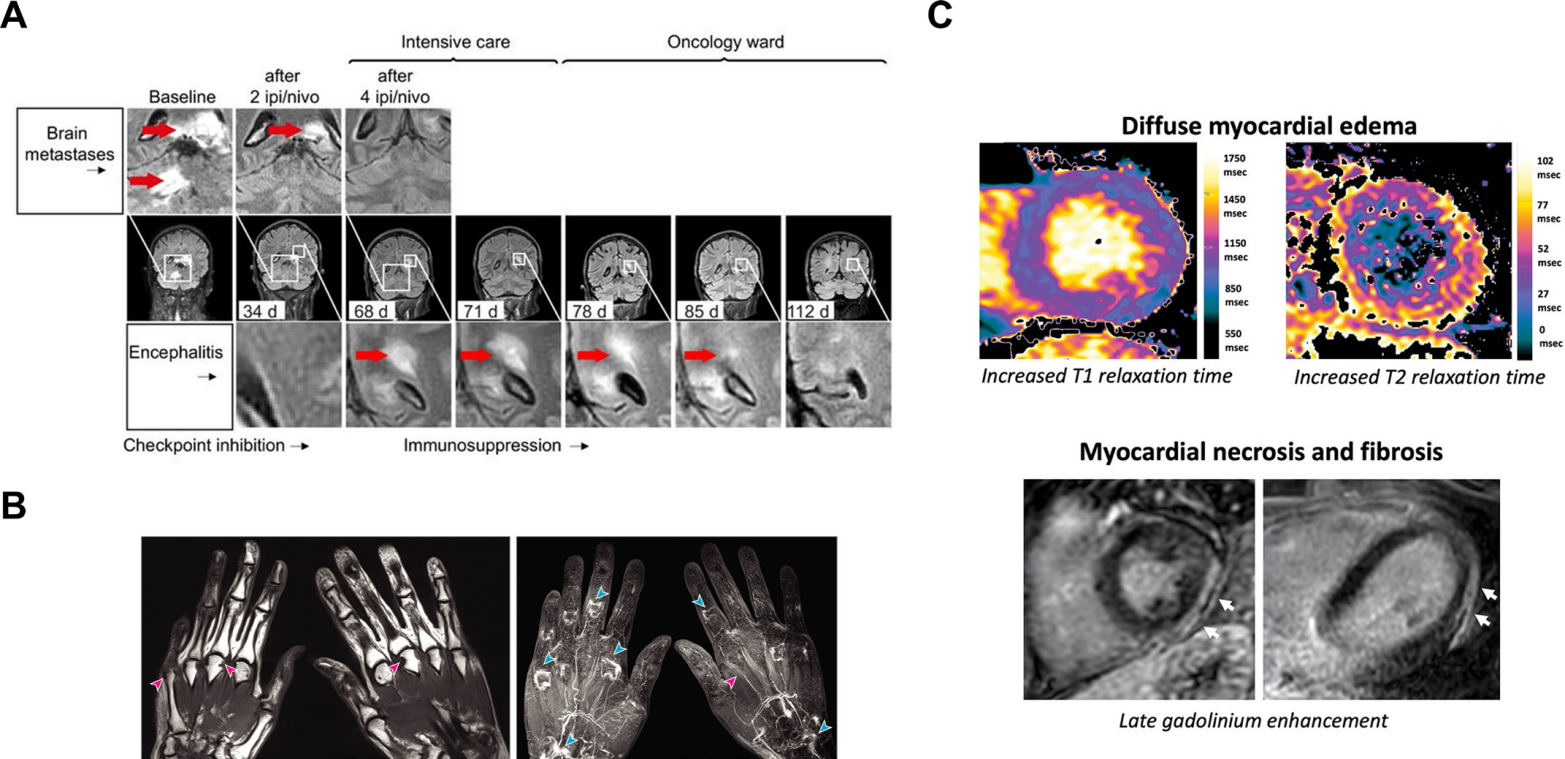
MRI of immune-related adverse events associated with immune checkpoint blockade. (A) T2 FLAIR imaging showed the simultaneous reduction of melanoma brain metastases and development of new diffuse inflammatory lesions (bright signal indicated by red arrow) in the brain, brain stem, and cerebellum of a patient who developed encephalomyelitis following two cycles of combined ipilimumab (ipi) and nivolumab (nivo). The inflammatory lesion in the brain was shown to resolve over time following temporary cessation of ICI treatment and 10 days of high-dose steroids. (B) T_1_-weighted imaging revealed multiple marginal bony erosions (pink arrow) and synovial enhancement following intravenous gadolinium-based contrast administration at the metacarpophalangeal, proximal interphalangeal, and intercarpal joints (blue arrows), and tenosynovitis (pink arrows), in both hands of a patient with ICI-associated inflammatory arthritis. (C) Increased T1 and T2 relaxation times associated with myocardial inflammation and late gadolinium enhanced lesions (white arrow) indicating myocardial necrosis and fibrosis as a latent effect of ICI-associated myocarditis. Images reproduced with permission from Bjursten *et al*, Subedi *et al*, and Faron *et al* 142 147 148. FLAIR, fluid-attenuated inversion recovery; ICI, immune checkpoint inhibitors.

As a technique, MRI provides excellent soft tissue contrast and anatomical resolution and has many applications in evaluating response to ICIs. It is a valuable tool for monitoring many of the neurological, gastrointestinal, rheumatological and cardiological manifestations of irAEs, some of which are shown in figure 4.^141–148^ For example, MRI can be used to detect encephalomyelitis associated with combined nivolumab and ipilimumab therapy.^142^ Inflammation can be detected as high signal on T_2_-weighted or fluid-attenuated inversion recovery (FLAIR) imaging due to the presence of increased water or ^1^H in edema, and enhancement on T_1_-weighted imaging following the administration of gadolinium-based contrast agents due to increased blood flow. The multiparametric nature of MRI can be used to distinguish the appearances of treatment-related irAEs from background tumor regression: figure 4a shows the development of new diffuse bilateral inflammatory brain lesions in a patient with metastatic melanoma involving the brain, indicating acute disseminated encephalomyelitis which required intensive care treatment, despite complete regression of the brain metastases after two treatment cycles.^142^ A gradual decrease in the inflammatory lesions in the brain and spinal cord was detected on MRI following 10 days of high-dose steroids and ICI treatment was resumed after the inflammatory lesions were shown to have completely resolved on MRI.

While central nervous system irAEs are rare, inflammation occurring in abdominal organs including colitis and hepatitis are some of the most common immunotoxicities. Early diagnosis, interruption of immunotherapy, and intervention with immunosuppressants where appropriate are needed to avoid morbidity and mortality. Colitis has been reported in 5–25% of patients treated with ICIs and is typically diagnosed with CT.^149^ MRI features of colitis include intestinal wall thickening, mural hyperenhancement, pericolic fat stranding, mesenteric hyperemia, with fluid-filled loops of bowel and its anatomical distribution can help distinguish this from other causes of diarrhea.^143^ DWI and DCE-MRI are less commonly used, but may help in the assessment of non-ICI-related colonic conditions, with increased cellular infiltration and perfusion in inflammatory bowel disease.^144 145^ In contrast to the clinical symptoms associated with colitis, hepatotoxicity is usually clinically silent and is initially detected on liver function tests. While international guidelines recommend liver biopsies in patients with severely deranged liver blood tests, at this stage steroids have usually been implemented already and the risks associated with this invasive procedure mean that liver biopsies are in fact rarely undertaken. This has hampered the understanding of the etiology of ICI-induced liver damage and in determining its optimal treatment. Multiparametric MRI including contrast enhancement is an important tool for evaluating new and evolving diffuse hepatic changes which complements and could replace the need for liver biopsy. MR elastography examining tissue stiffness may be used for evaluating liver fibrosis from acute liver injury in inflammatory conditions.^146^ These methods could have a role in differentiating drug-induced changes superimposed on a background of evolving hepatic metastases.^143^

Rheumatoid or musculoskeletal irAEs such as myofascitis and inflammatory arthritis are common chronic problems linked to ICIs^150 151^ which may often persist after treatment cessation.^152^ MRI is superior to CT and ultrasound for evaluating musculoskeletal inflammation, enabling the detection of synovitis in the joints with inflammatory arthritis, and changes in volume and contrast enhancement of the synovium following anti-inflammatory drug treatment.^153^ It is a sensitive method for the detection and quantification of bone erosions and bone marrow edema associated with inflammatory arthritis.^154 155^ Irregular synovial thickening and tenosynovitis were shown to be early radiological features of inflammatory arthritis, even in patients with minimal symptoms.^147 156^ Bone erosions, synovial enhancement, and tenosynovitis have also been detected on contrast-enhanced MRI in a subset of patients with more aggressive inflammatory arthritis.^147^

ICI-related myocarditis is a very rare but potentially fatal event that can occur with ICIs.^157^ Multiparametric cardiac MRI has been used as a means of detecting myocardial inflammation and systolic dysfunction in a prospective trial on 22 patients with no pre-existing cardiac conditions following 3 months of ICI treatment.^148^ The cardiac MRI protocol used myocardial strain analysis to evaluate systolic function, and T_1_-mapping and T_2_-mapping to evaluate myocardial edema and late gadolinium enhancement (LGE) imaging to assess myocardial fibrosis. A decrease in left ventricular ejection fraction indicating systolic dysfunction was detected at follow-up on MRI and the T_1_ and T_2_ relaxation times were found to increase in patients with diffuse myocardial edema. Pericardial and pleural effusions were more frequently detected at the follow-up scans compared with baseline. New focal LGE lesions in a non-ischemic distribution were detected in two patients, possibly caused by acute inflammatory necrotic lesions. These demonstrated the utility of multiparametric MRI in monitoring microstructural and functional changes in cardiac tissue following ICI.

### Hybrid PET/MRI in immunotherapy

The concept of using hybrid PET/MRI imaging for immunotherapy monitoring is relatively underexplored but offers great potential for phenotyping and imaging of immune responses following treatment by exploiting the distinct benefits of each modality.^158^ Immunotherapy-induced changes in tumors and non-target tissues (which may be the cause of irAEs) are complex processes that may be best probed by a multimodality approach. PET/MRI combines the high spatial resolution and unique tissue characterization afforded by MRI with the high molecular sensitivity of PET which provides a wide range of radiotracers to phenotype the tumor immune microenvironment. Combining multiparametric MRI with [^18^F]FDG-PET in a clinical setting could provide a wealth of information for improved understanding of response to immunotherapy than each modality alone, informing on the tissue structure, function, and immunometabolism in a single examination protocol.^159 160^ Tumor heterogeneity and vascular permeability may affect immunotherapeutic drug delivery, and this can be explored with PET/MRI by combining DKI and DCE-MRI image acquisition with PET imaging of radiolabeled drugs. In the experimental setting, vascular inflammation and recruitment of leukocytes can be simultaneously examined by combining antibody-conjugated MPIO imaging (eg, targeting ICAM-1)^58^ with immune-specific PET probes (eg, targeting CD8)^27^ or labeled cell tracking techniques on PET.^93^ The interplay between tumor cell death in response to treatment and immunosuppressive mechanisms such as tumor acidity, metabolic aberrations, and the expression of immune checkpoint proteins or other exhaustion markers can potentially be examined in the future within a single imaging session using DWI,^5^ HP ^13^C-MRI^87^ and PET probes such as those targeting PD-1 and PD-L1.^29^

The [^18^F]FDG PET/MRI has been investigated as a hybrid approach for predicting early response to ICIs in patients with non-small cell lung carcinoma and melanoma.^161 162^ A combination of measurements on the changes in total lesion glycolysis (TLG) derived from [^18^F]FDG uptake and ADC measurement of tumor cellularity on DWI (ΔTLG+ΔADC_mean_) before and after 2 weeks of nivolumab therapy was found to be predictive of response and survival in patients with non-small cell lung carcinoma.^161^ ΔTLG+ΔADC_mean_ demonstrated higher sensitivity, specificity, and accuracy than size measurement alone, or individual PET or MRI metrics, and was found to be particularly useful in evaluating metabolic pseudoprogression in early response to treatment. In an exploratory study on a small number of patients with glioblastoma, MRI was used in combination with PET imaging of deoxycytidine kinase (an enzyme overexpressed in leukocytes and some cancers) using the PET radiotracer, 2-chloro-2’-deoxy-2’-[^18^F]fluoro-9-b-*D*-arabinofuranosyl-adenine ([^18^F]CFA). A multiparametric MRI protocol involving contrast-enhanced MRI and DWI showed potential in detecting changes in cellularity and tumor perfusion associated with [^18^F]CFA uptake and immune infiltration in patients responding to treatment with a DC vaccine and: the superior soft tissue contrast obtained using MRI facilitated the detection of [^18^F]CFA uptake in tumors and lymph nodes.^163^ A recent first-in-human study investigated *ex vivo* labeling of CAR T cells targeting the carbohydrate Lewis Y antigen using Copper-64 labeled SPIO nanoparticles: these labeled cells were reinfused into patients with solid tumors and imaged using PET/MRI within 3–5 days to track the distribution of labeled cells to tumors and other body organs.^164^ The addition of a PET label facilitated sensitive detection of the cells and MRI imaging enabled detection of the SPIO label while providing excellent delineation of soft tissue structures including the peripheral lymph nodes.

However, at the moment, PET/MRI is not as widely adopted as PET/CT due to the high capital cost of the scanner and longer acquisition times.^158^ Optimization of MRI sequences may help to address the issues with long acquisition times such as the use of parallel imaging, compressed sensing techniques, abbreviated protocols, or MR fingerprinting techniques.^158 165–168^ Furthermore, improvements have been made to overcome the technical challenges in attenuation correction for PET/MRI images using atlas-based approaches and machine learning methods to generate pseudo-CT attenuation maps.^169^

## Conclusions

MRI has a major role to play in evaluating the tumor immune environment in the preclinical setting, and as part of experimental medicine studies, while its value in routine clinical practice is increasing. This review has described its potential as a tool to better predict short-term and long-term response to immunotherapy, and its importance in the diagnosis and management of a variety of irAEs. The strengths of MRI include the detailed soft tissue anatomy it provides, the wide range of contrast mechanisms it can exploit using multiparametric imaging, the ability to non-invasively discriminate metabolites, and the absence of ionizing radiation. Several MRI approaches have been used to probe the immune microenvironment and its response to immunotherapy including changes in celldensity, tissue microstructure, tumor vascularity, cellular infiltration, and tissue metabolism. The major limitation of MRI is its lack of sensitivity when compared with radionuclide imaging, but the advent of hybrid PET/MRI has generated opportunities to combine the strengths and compensate the limitations of both modalities in a single imaging session, with greater clinical application likely to become evident in the coming years.

10.1136/jitc-2022-004708.supp1 Supplementary data
