# Supplementary material for: MRI techniques for immunotherapy monitoring
Source: J Immunother Cancer. 2022 Sep 19;10(9):e004708. doi: 10.1136/jitc-2022-004708 (PMC9486399; doi:10.1136/jitc-2022-004708)
Supplement: Supplementary data [file jitc-2022-004708supp002.pdf]

Supplemental Table S1. Overview of MRI techniques used in monitoring cancer immunotherapy.

Biological Phenomenon	MRI Techniques	Application	Stage of Testing	References
**Tissue characterization**	T_1-weighted MRI<br>T_2-weighted MRI	Tissue characterization	Clinical	5, 39
**Tumor Cellularity**	DWI, IVIM, DKI	Tumor cell death or cell loss following immune checkpoint blockade, dendritic cell vaccination, and adenoviral gene therapy	Clinical	5, 46, 48, 49
**Vascular Permeability and Inflammation**	Contrast enhanced MRI	Qualitative assessment of tissue vascularity	Clinical	40
	DCE-MRI	Quantitative measures of vascular permeability following immune checkpoint blockade	Clinical	5, 54
	Antibody-conjugated MPIO e.g. VCAM-1	Vascular inflammation following PD-L1 monotherapy	Preclinical	56
**Tumor Metabolism**	^{1}H-MRS	*In vivo* assessment of metabolites	Clinical	73
	CEST MRI	Measurement of targeted metabolites	Clinical	74
	Hyperpolarized ^{13}C-MRI			
	[1-^{13}C]pyruvate	Glycolysis	Preclinical / Clinical	84, 85, 86
	[1,4-$^{13}C_2$]fumarate	Cell Death	Preclinical	85

**Tracking Labeled Leukocytes**	SPIO	Therapies targeting tumor-associated macrophages e.g. anti-CD47 monoclonal antibodies	Preclinical / Clinical	96, 97, 98, 99
	^{19}F-PFC	Cell-based therapies e.g. T cells, NK cells, DCs	Preclinical / Clinical	107, 108, 109
**Reporter Gene Imaging**	Lysine-rich protein	Oncolytic viral therapy	Preclinical	122
	Ferritin	Dendritic cell vaccination	Preclinical	123
	Nucleoside kinase	Dendritic cell vaccination	Preclinical	124
**Immune-related Adverse Events**	T_1-weighted MRI<br>T_2-weighted MRI<br>T_1/T_2-mapping<br>Late gadolinium enhancement<br>DCE-MRI<br>DWI<br>MRE	Encephalomyelitis	Clinical	141
		Colitis	Clinical	142, 143, 144
		Hepatitis	Clinical	145
		Inflammatory Arthritis	Clinical	146
		Myocarditis	Clinical	147
**Hybrid Imaging**	DWI + [^{18}F]FDG PET (Tumor cellularity + glucose metabolism)	Immune checkpoint inhibitors	Clinical	160, 161
	T_2-weighted MRI, Contrast enhanced T_1-weighted MRI, DWI + [^{18}F]CFA PET	Dendritic cell vaccination	Preclinical / Clinical	162

(Tumor cellularity and vascularity + deoxycytidine kinase activity)			
[64]Cu labeled SPIO nanparticles for cell tracking	CAR T cell therapy	Preclinical	163

MRI = magnetic resonance imaging; DWI = diffusion-weighted imaging; IVIM = intravoxel incoherent motion; DKI = diffusion kurtosis imaging; DCE-MRI = dynamic contrast-enhanced MRI; MPIO = microparticles of iron oxide; MRS = magnetic resonance spectroscopy; CEST MRI = chemical exchange saturation transfer (CEST); SPIO: superparamagnetic iron oxide; ^{19}F-PFC = Fluorine-19 perfluorocarbon; PET = positron emission tomography; [^{18}F]FDG = Fluorine-18 fluorodeoxyglucose; [^{18}F]CFA = 2-chloro-2'-deoxy-2'-[^{18}F]fluoro-9-b-D-arabinofuranosyl-adenine.

N.B. The MRI techniques described in this review were updated till July 2022. The focus is on clinical application and highlighting MRI methods investigated in cancer immunotherapy.
