# Supplementary material for: MRI techniques for immunotherapy monitoring
Source: J Immunother Cancer. 2022 Sep 19;10(9):e004708. doi: 10.1136/jitc-2022-004708 (PMC9486399; doi:10.1136/jitc-2022-004708)
Supplement: Supplementary data [file jitc-2022-004708supp003.pdf]

Supplemental Table S2.

List of abbreviations for the technical terms described in this review.

Abbreviation	Technical Term
CEST	Chemical exchange saturation transfer
CMR	Complete metabolic response
CT	Computed tomography
D_{app}	Apparent diffusivity
Dm-dNK	Drosophila melanogaster 2'-deoxynucleoside
DCE-MRI	Dynamic contrast-enhanced MRI
DWI	Diffusion-weighted imaging
DKI	Diffusion kurtosis imaging
EPR	Enhanced permeability and retention effects
[^{18}F]CFA	2-chloro-2'-deoxy-2'-[^{18}F]fluoro-9-b-*D*-arabinofuranosyl-adenine
[^{18}F]FDG	Fluorine-18 fluorodeoxyglucose
[^{18}F]FLT	Fluorine-18 fluorothymidine
^{19}F-PFC	Fluorine-19 perfluorocarbon
FLAIR	Fluid-attenuated Inversion Recovery
FTH	Ferritin
Gd-EOB-DTPA	Gadolinium ethoxybenzyl diethylenetriamine pentaacetic acid
HP ^{13}C-MRI	Hyperpolarized Carbon-13 MRI
ICI	Immune checkpoint inhibitor
irAEs	Immune-related adverse events
iRANO	Immune Response Assessment in Neuro-Oncology
irRC	Immune-related Response Criteria
K_{app}	Apparent kurtosis
K^{trans}	Volume transfer constant from the blood plasma to the extravascular tumor interstitial space
LAG3	Lymphocyte activation gene 3
LDH	Lactate dehydrogenase
LGE	Late gadolinium enhancement
LRP	Lysine-rich protein
MPIO	Microparticles of iron oxide
MRI	Magnetic resonance imaging
MRS	Magnetic resonance spectroscopy
OATP	Organic anion transporting polypeptide

PET	Positron emission tomography
pyrrolo-dC	pyrrolo-2’-deoxycytidine
RECIST	Response Evaluation Criteria in Solid Tumors
SPECT	Single photon emission computed tomography
SPIO	Superparamagnetic iron oxide
T1	Spin-lattice or longitudinal relaxation time
T2	Spin-spin or transverse relaxation time
TLG	Total lesion glycolysis
USPIO	Ultrasmall superparamagnetic iron oxide
v_e	Fractional volume of the interstitial space
v_p	Fractional volume of the blood plasma
