# Supplementary figures and images for: MRI techniques for immunotherapy monitoring
Source: J Immunother Cancer. 2022 Sep 19;10(9):e004708. doi: 10.1136/jitc-2022-004708 (PMC9486399; doi:10.1136/jitc-2022-004708)

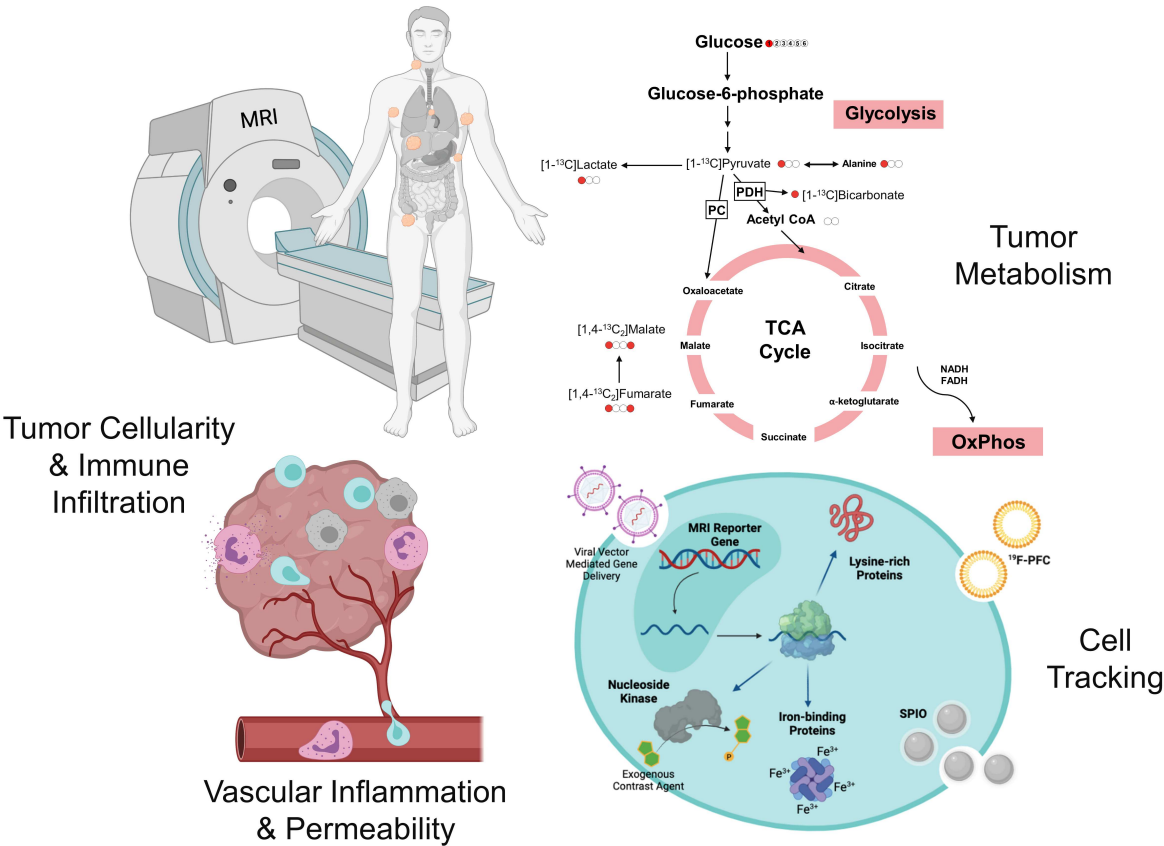

Supplement: Supplementary data [file jitc-2022-004708supp001.pdf]
